# Trajectory changes of symptom clusters in patients with concurrent chemoradiotherapy for cervical cancer: a prospective longitudinal study

**DOI:** 10.3389/fonc.2026.1808208

**Published:** 2026-05-18

**Authors:** Yuejia Wang, Xue Jin, Shuchi Zhao, Zixuan Zhang, Lamei Liu, Xiangyu Lu, Shengwu Wu, Lingling Zheng, Zuochuan Wang, Yin Lv, Yingge Lei, Hua Du

**Affiliations:** 1 School of Nursing, Anhui Medical University, Hefei, China; 2 Department of Radiation Oncology, The First Affiliated Hospital of Anhui Medical University, Hefei, China; 3 Outpatient Operating Room II, The First Affiliated Hospital of Anhui Medical University, Hefei, China

**Keywords:** cervical cancer, concurrent chemoradiotherapy, group trajectory model, longitudinal study, symptom clusters

## Abstract

**Objective:**

To identify the trajectory changes of symptom clusters during concurrent chemoradiotherapy in cervical cancer patients and to explore the heterogeneity of symptom experience among different patients.

**Methods:**

From June 2024 to June 2025, 150 patients with cervical cancer undergoing concurrent chemoradiotherapy at a tertiary-level hospital in Anhui Province were selected as the study subjects. A general information questionnaire and the Symptom Assessment Scale for Patients with Intermediate-Advanced Cervical Cancer Undergoing Concurrent Chemoradiotherapy were used to conduct a questionnaire survey at the end of 10 radiotherapy sessions (T1), the end of 20 radiotherapy sessions (T2), the end of radiotherapy (T3), and 3 months after the end of radiotherapy (T4). The Group-based Trajectory Model (GBTM) was used to identify the trajectory changes of symptom clusters during concurrent chemoradiotherapy.

**Results:**

This study found that the symptom trajectories during concurrent chemoradiotherapy for cervical cancer could be divided into four categories: low symptom burden group (n=77, 51.33%), moderate symptom burden group (n=16, 10.67%), high symptom burden group (n=36, 24%), and moderate symptom burden - delayed symptom group (n=21, 14%). There were statistically significant differences among the four categories in terms of disease duration, surgical history, and presence of other chronic diseases (*P* < 0.05).

**Conclusion:**

Healthcare professionals should closely monitor symptom changes in cervical cancer patients undergoing concurrent chemoradiotherapy, identify symptom clusters early, and implement individualized and precise symptom management strategies.

## 1 Introduction

As the fourth most common cancer among women worldwide, cervical cancer seriously threatens women’s health and lives. In 2022, there were approximately 660,000 new cases of cervical cancer and approximately 350,000 deaths worldwide (1). In the same year, there were approximately 150,000 new cases of cervical cancer and approximately 60,000 deaths in China, accounting for 22.7% and 16% of the global incidence and mortality rates respectively (2). Domestic and international guidelines show that the synergistic effect of concurrent chemoradiotherapy can maximize the anti-tumor effect and is an important means of treating cervical cancer (3, 4). However, concurrent chemoradiotherapy is prone to causing a series of physiological and psychological discomfort symptoms, such as persistent fatigue, pain, anxiety, gastrointestinal dysfunction and sexual dysfunction (5). These symptoms usually appear in clusters, and Dodd (6) defined these interrelated and simultaneous symptoms as symptom clusters. Longitudinal studies of symptom clusters focus on the dynamic changes of symptoms and can help clinicians develop predictive symptom management strategies.

The Group-based Trajectory Model (GBTM) adopts a semi-parametric trajectory modeling strategy. Its core assumption is that the population consists of several potential growth trajectory subgroups. The model calculates the posterior probability of each individual belonging to each potential trajectory and uses the growth curve corresponding to the maximum posterior probability as the basis for subgroup affiliation, thereby achieving population clustering. Each potential growth curve represents a subgroup. Different subgroups have different development patterns, while all individuals within the same subgroup follow the same growth trajectory pattern and intercept parameter. This model makes up for the shortcomings of traditional growth models in exploring population heterogeneity (7). GBTM can identify different development trajectories in the population and estimate the probability of an individual belonging to a trajectory group. It can predict future clinical events based on known data, thereby achieving dynamic prediction of the patient’s disease development trajectory and expected results (8).

Research on the symptomology of cervical cancer, both domestically and internationally, is still in its early stages. The main approach is to conduct cross-sectional surveys and explore the relationship between symptom groups and quality of life. Xia Weishu (9) used the Anderson Symptom Assessment Scale to investigate the frequency and severity of symptoms during concurrent chemoradiotherapy in 200 cervical cancer patients. He found that fatigue was the most common and sadness was the most severe. He also extracted four symptom groups: emotional symptom group, gastrointestinal symptom group, treatment adverse reaction symptom group and fatigue symptom group. All dimensions of quality of life were significantly negatively correlated with the four symptom groups. Pelizzola (10) investigated the symptom groups of 742 patients with locally advanced cervical cancer after chemoradiotherapy. He identified three organ-related symptom groups (urinary system, gastrointestinal tract, vagina), including a comprehensive symptom group with symptoms such as fatigue, pain and insomnia, and a symptom group including nausea, vomiting and loss of appetite. The gastrointestinal symptom group, comprehensive symptom group and nausea symptom group were significantly associated with the overall quality of life of patients. At present, the trajectory of symptom group changes during concurrent chemoradiotherapy in cervical cancer patients is still unclear. This study aims to identify the symptom patterns of cervical cancer patients during concurrent chemoradiotherapy using a group trajectory model, helping medical staff predict changes in patients’ symptoms and providing a theoretical basis for developing effective interventions to improve the quality of life of cervical cancer patients after treatment.

## 2 Materials and methods

### 2.1 Patients and treatment

Convenience sampling was used to select cervical cancer patients who underwent concurrent chemoradiotherapy at a radiotherapy center of a tertiary-level hospital in Anhui Province from June 2024 to June 2025 as the study subjects. Inclusion criteria: (1) Cervical cancer patients diagnosed by histopathology; (2) Age > 18 years, able to communicate normally and cooperate with the researchers to complete the questionnaire; (3) Informed consent and voluntary participation in the survey; (4) Patients whose treatment was concurrent chemoradiotherapy. Concurrent chemoradiotherapy treatment regimen: The total dose of external beam radiation therapy was 50–56 Gy, 1.8–2 Gy/fraction, 5 times a week, for a total of 25–28 fractions, completed in about 5–6 weeks; intracavitary brachytherapy was interspersed during the period, with a total dose of 20–25 Gy, for a total of 4–5 fractions. The chemotherapy regimen was platinum-based (mainly cisplatin) single-agent or combination chemotherapy. Exclusion criteria: (1) Patients with mental illness or cognitive impairment; (2) Patients with other tumors or major diseases.

This study is a prospective longitudinal study. The sample size calculation formula (11) was used: N=(U_a/2_S/δ)^2^, where α=0.05, U_α/2_ = 1.96. Based on the review of previous studies, x̅=53.16, S = 14.51, and the allowable error was controlled at 5% of the mean: δ=2.658. The required sample size for this study was approximately 115 cases. Considering that this study is a longitudinal study and has a certain sample attrition rate, it is proposed to increase the sample size by 20%, and the final sample size is 138 cases.

### 2.2 Measurements

#### 2.2.1 General information questionnaire

Designed by the researchers themselves, the study included general demographic information of patients (age, education level, occupation, number of children, marital age, religious beliefs, living environment, average monthly income per capita in the family, medical expense payment methods.) and information related to the patient’s disease and treatment (tumor type, tumor stage, whether surgery was performed, whether there were other chronic diseases.).

#### 2.2.2 Symptom assessment scale for patients with intermediate-late stage cervical cancer undergoing concurrent radiotherapy and chemotherapy

Symptom assessment was performed using the Symptom Assessment Scale for Patients with Intermediate-Advanced Cervical Cancer Undergoing Concurrent Radiotherapy and Chemotherapy, developed by Zhang Renjing (12). The scale consists of 23 items across 6 dimensions: psychological symptoms (including anxiety, sadness, tension, low mood, and irritability); nutritional symptoms (including nausea, vomiting, decreased appetite, and weight loss); intestinal symptoms (including tenesmus, anal pain, diarrhea, abdominal pain, and constipation); urinary symptoms (including urinary urgency, frequency, and burning pain during urination); sexual symptoms (including loss of interest in sex and fear of sex); and somatic symptoms (including poor sleep, numbness in hands and feet, generalized or localized pain, and fatigue). A 5-point Likert scale is used, with scores from 1 to 5 representing “none,” “mild,” “moderate,” “severe,” and “major,” respectively. A higher sum of scores indicates more severe symptoms. The scale’s Cronbach’s α coefficient was 0.813, its split-half reliability was 0.853, and its test-retest reliability was 0.998. In this study, the scale’s Cronbach’s α coefficient ranged from 0.723 to 0.856.

### 2.3 Data collection and quality control

The general information questionnaire for this study was obtained upon patient admission. The cervical cancer symptom assessment scale was used at the end of 10 radiotherapy sessions (T1), the end of 20 radiotherapy sessions (T2), the end of radiotherapy (T3), and three months after the end of radiotherapy (T4). Data collectors, trained researchers, distributed questionnaires to eligible patients undergoing concurrent chemoradiotherapy for cervical cancer and provided guidance on completion. Before completion, the purpose and content of the study were explained to patients and their families. With patient consent, the survey on general information and the occurrence of symptoms during concurrent chemoradiotherapy for cervical cancer was conducted. If patients were unable to complete the questionnaire themselves, the data collectors read each item aloud and recorded the patient’s answers objectively and accurately. Questionnaires were collected and checked on-site, and any omissions were promptly corrected. Patients were informed that data would be collected multiple times during treatment and follow-up examinations, and the contact information of the patient or their primary caregiver was retained. If subsequent on-site completion of the questionnaire was not possible, data was collected online via telephone or other means.

### 2.4 Statistical considerations

Data were entered using EpiData 3.1 by two individuals and described statistically using IBM SPSS Statistics 26.0. Count data were expressed as n (%), and symptom severity scores that did not conform to a normal distribution were expressed as M (P25, P75). (x̅ ± s) was used as an auxiliary evaluation tool. Trajectory recognition was performed using the gbmt software package in R4.5.1.: Missing values for the six symptom cluster variables from T1 to T4 were imputed using the LOCF method to obtain balanced panel data. Subsequently, GBMT models with 1 to 6 categories were fitted sequentially. Each model described the trajectory of each symptom over time using a cubic polynomial, and log-likelihood, information criterion, and prior probability were extracted. The traditional likelihood ratio test for adjacent category models was calculated based on log-likelihood, and the chi-square distribution was used to determine whether increasing the number of categories significantly improved the fit. The GBTM model fit indices mainly included the Akaike Information Criterion (AIC), Bayesian Information Criterion (BIC), and Sample-Size Adjusted Bayesian Information Criterion (SSBIC) to evaluate the model fit; the smaller the value, the better the fit. The Consistent Akaike Information Criterion (CAIC) and Hannan-Quinn Information Criterion (HQIC) were used for auxiliary evaluation. The actual explanatory index is judged based on clinical experience to determine whether the trajectory model obtained through GBTM conforms to reality (13, 14). The Entropy index evaluates the classification accuracy, with a value ranging from 0 to 1. The larger the value, the more accurate the classification. A value greater than 0.8 indicates a classification accuracy of over 90%.

## 3 Results

### 3.1 Survey of follow-up status

**Figure 1:**
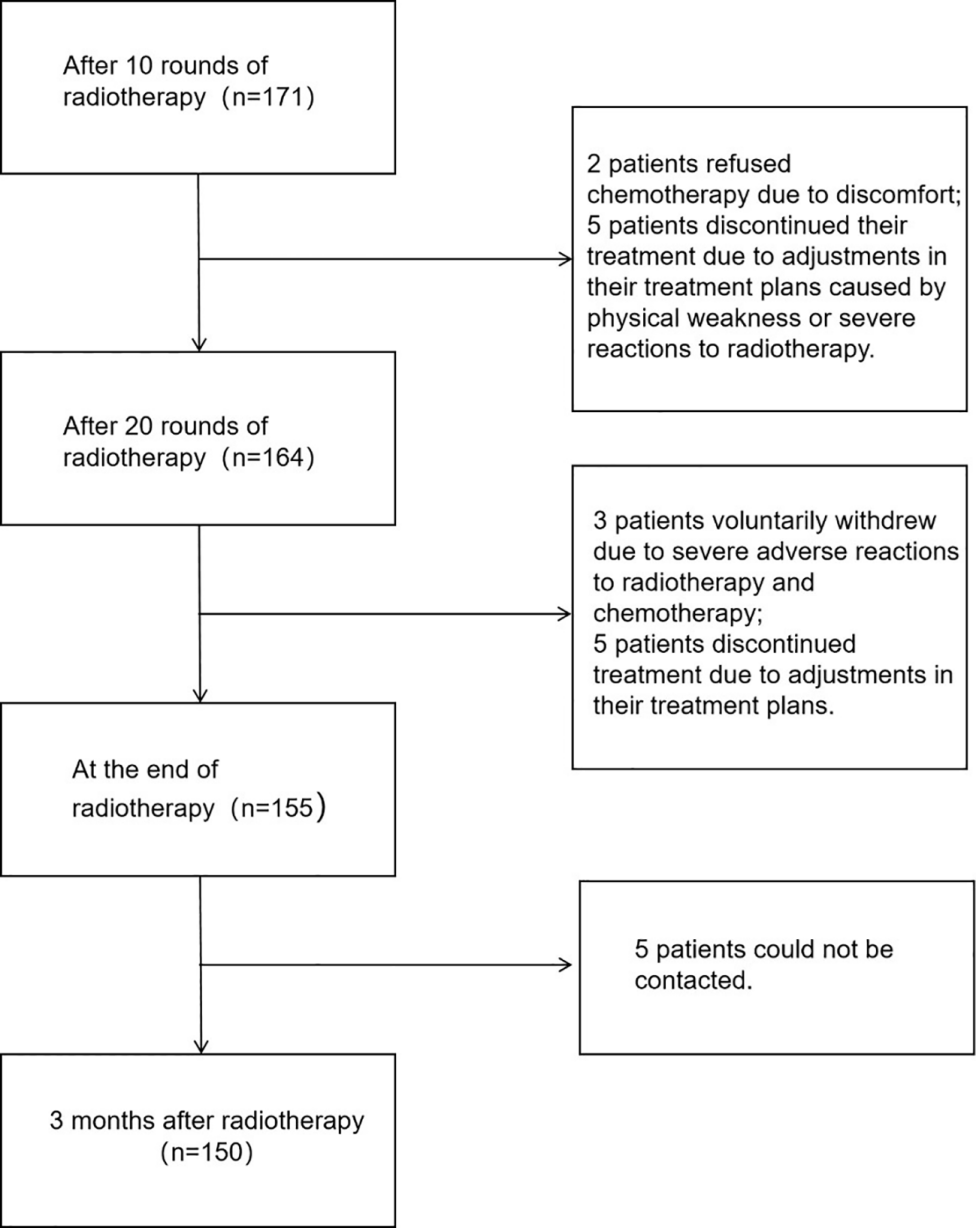
Patient follow-up status.

In this study, 171 questionnaires were collected before 10 sessions of radiotherapy; 164 questionnaires were collected before 20 sessions of radiotherapy; 155 questionnaires were collected after the end of radiotherapy; and 150 questionnaires were collected 3 months after the end of radiotherapy, with an overall loss to follow-up rate of 12.28%. See **Figure 1** for specific screening results.

### 3.2 General information about cervical cancer patients

The average age of the patients was (57.51 ± 10.69) years; all 155 patients were married, with 119 having been married for more than 20 years and 83 having two or more children; 10 patients had religious beliefs; 21 patients lived in rural areas, 30 in townships, and 89 in cities; the majority of families had a monthly per capita income of 2,000-5,000 yuan, and medical expenses were mainly paid through resident medical insurance. Tumor types: squamous cell carcinoma (122 cases), adenocarcinoma (13 cases), and other types (5 cases); tumor stages: stage I (27 cases), stage II (46 cases), stage III (59 cases), and stage IV (8 cases); 80 patients underwent postoperative concurrent chemoradiotherapy; 42 patients had other chronic diseases. See **Table 1** for details.

**Table 1:** General demographic data and disease- and treatment-related data of patients undergoing concurrent chemoradiotherapy for cervical cancer (n=150).

Classification	Examples(n)	Composition ratio(%)
Age		
≤40	15	10.0
41~50	17	11.3
51~60	64	42.7
>60	54	36.0
Educational level		
Primary school and below	96	64.0
Junior high school	37	24.7
High school or vocational school	10	6.7
College and above	7	4.7
Occupation		
Farmer	41	27.3
Worker	8	5.3
Enterprise employee	4	2.7
Retired people	9	6.0
Freelance work	11	7.3
Unemployed	77	51.3
Number of children		
0	6	4.0
1	56	37.3
≥2	88	58.7
length of marriage(year)		
≤10	6	4.0
11-20	16	10.7
>20	128	85.3
Religion		
No	140	93.3
Yes	10	6.7
Residence		
Rural	26	17.3
Villages and towns	32	21.3
Urban	92	61.3
Monthly household income(RMB)		
<2000	31	20.7
2000-5000	102	68.1
>5000	17	11.3
Medical expense payment method		
Employee medical insurance	7	4.7
Residents’ medical insurance	142	94.7
Commercial insurance	1	0.7
Tumor type		
Squamous carcinoma	130	86.7
Adenocarcinoma	13	8.7
Other tumors	7	4.7
Clinical stage		
I	28	18.7
II	49	32.7
III	63	42.0
IV	10	6.7
Course of illness(month)		
<3	24	16.0
3~6	111	74.0
>6	15	10.0
History of surgery		
Yes	87	58.0
No	63	42.0
Cobine with other chronic diseases		
Yes	87	58.0
No	63	42.0

### 3.3 Symptom trajectory changes in cervical cancer patients during concurrent chemotherapy and radiotherapy

This study fitted a total of 6 models, and the specific fitting results are shown in **Table 2**. Fitting began with the first model, and the number of classes fitted was increased sequentially. The final model was determined by combining model indicators with clinical practice. As the number of classes increased, AIC and BIC gradually decreased, with AIC reaching its lowest value in Model 5 and BIC in Model 4. The Entropy values of the two models were 0.989 and 0.988, respectively, indicating high classification clarity and good class differentiation. When Entropy values are similar, models with fewer classes, greater simplicity, and no extreme distributions are preferred. Although the AIC of Model 5 was slightly lower than that of Model 4, BIC, CAIC, and HQIC all increased, indicating overfitting. Furthermore, in Model 5, class 2 accounted for only 2.67%, resulting in a small sample size that could lead to unstable trajectory estimation and insufficient clinical generality. Model 4 had a relatively balanced distribution of the four classes (52.0%, 19.0%, 22.8%, and 18.3%), with sufficient samples supporting each class. Therefore, Model 4 was selected as the optimal trajectory model.

**Table 2:** Fitting indices for the symptom cluster trajectory model in cervical cancer patients undergoing concurrent chemoradiotherapy.

Model	AIC	BIC	CAIC	SSBIC	HQIC	Entropy	Posterior probability
1	-3894.885	-3705.817	-3662.817	-3842.331	-3842.331-	–	1
2	-4112.504	-3738.765	-3653.765	-4008.617	-3967.015	0.956	0.8290555/0.1709445
3	-10660.62	-10132.99	-10012.99	-10513.96	-10455.23	0.988	0.62/0.2403068/0.1396932
4	-11772.23	-11073.12	-10914.12	-11577.9	-11500.08	0.988	0.5199987/0.1905/0.2275/0.1825
5	-11838.91	-10946.34	-10743.34	-11590.81	-11491.45	0.989	0.5133333/0.0266667/0.1066667/0.2066279/0.1467055
6	-10694.29	-9683	-9453	-10413.19	-10300.62	0.992	-.6133333/0.0066667/0.0066667/0.1094676/0.1513037/0.1125621

**Figure 2:**
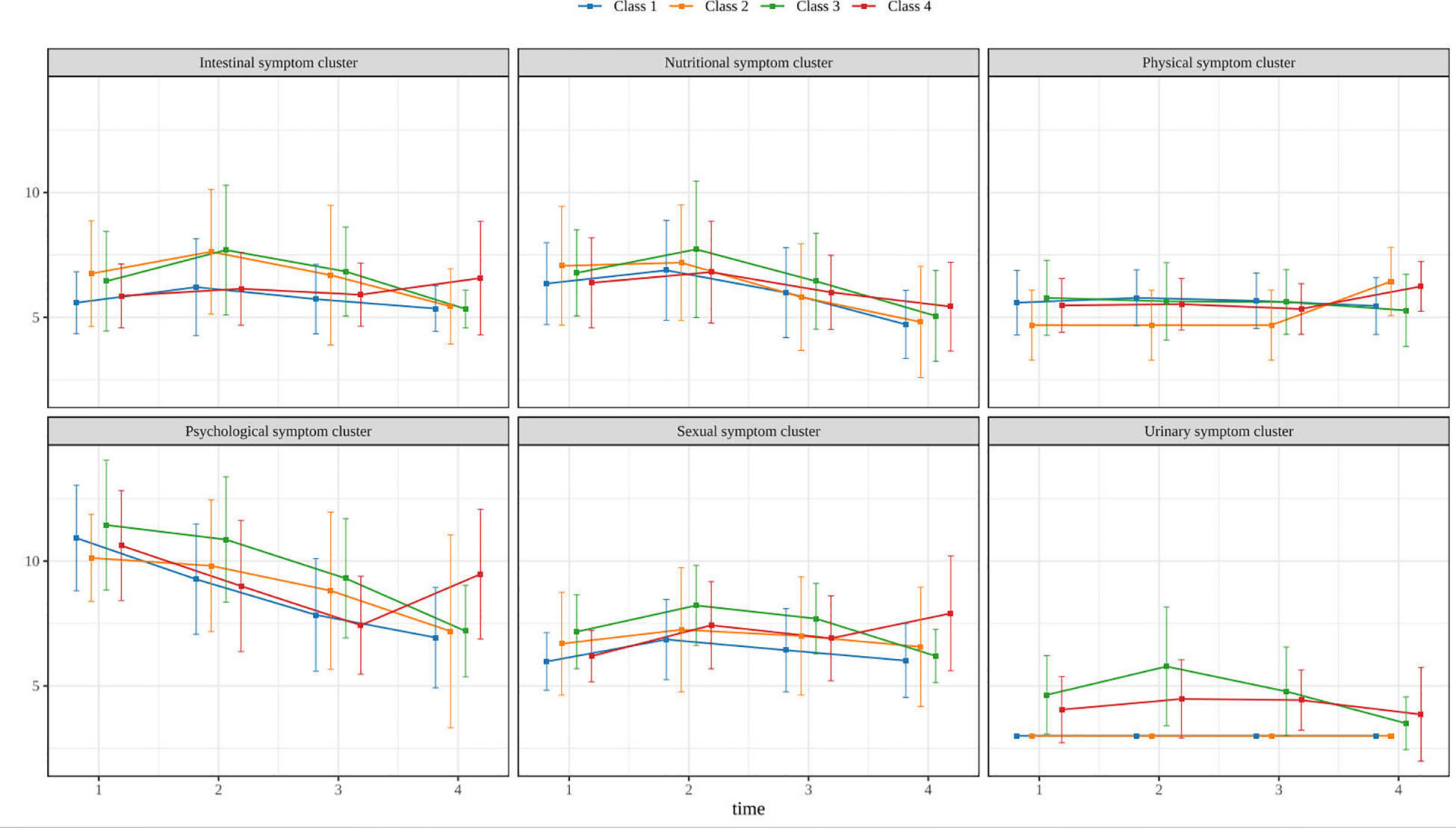
Trajectory of different symptom clusters during concurrent chemoradiotherapy in cervical cancer patients.

Based on Model 4, the changes in the symptom cluster trajectories of each group are shown in **Figure 2**, and the specific values are shown in **Table 3**. Patients in category 1 had scores in almost all symptom clusters at the lowest level, so this group was named the low symptom burden group. Patients in category 2 had symptom cluster scores that were basically similar to those in category 1, except for somatic symptom cluster 4, but the scores were slightly higher than those in category 1, so this group was named the medium symptom burden group. Patients in category 3 had scores in almost all symptom clusters at high values, so this group was named the high symptom burden group. Patients in category 4 typically had scores between the lowest and highest values for each symptom cluster, and the intestinal, somatic, psychological, and sexual symptoms clusters all showed an increase at T4, so this group was named the moderate symptom burden - delayed symptom group.

**Table 3:** Symptom cluster scores of different patient categories at different time periods.

Time	Symptom cluster	Low symptom burden group (n=77)	Moderate symptom burden group (n=16)	High symptom burden group (n=36)	Moderate symptom burden- delayed symptom group (n=21)
T1	psychological symptom cluster	10.83 ± 1.970	10.13 ± 1.746	11.53 ± 2.668	10.52 ± 2.182
	nutritional symptom cluster	6.35 ± 1.636	7.06 ± 2.380	6.78 ± 1.726	6.38 ± 1.802
	intestinal symptom cluster	5.58 ± 1.239	6.75 ± 2.113	6.44 ± 1.992	5.86 ± 1.276
	urinary system symptom cluster	3.00 ± 0.000	3.00 ± 0.000	4.64 ± 1.570	4.05 ± 1.322
	sexual life symptom cluster	5.58 ± 1.291	4.69 ± 1.401	5.78 ± 1.495	5.48 ± 1.078
	somatic symptom cluster	5.97 ± 1.158	6.69 ± 2.056	7.17 ± 1.483	6.19 ± 1.030
T2	psychological symptom cluster	9.27 ± 2.210	9.81 ± 2.639	10.86 ± 2.509	9.00 ± 2.627
	nutritional symptom cluster	6.88 ± 2.006	7.19 ± 2.316	7.72 ± 2.732	6.81 ± 2.040
	intestinal symptom cluster	6.21 ± 1.935	7.63 ± 2.500	7.69 ± 2.595	6.14 ± 1.459
	urinary system symptom cluster	3.00 ± 0.000	3.00 ± 0.000	5.78 ± 2.380	4.48 ± 1.569
	sexual life symptom cluster	5.78 ± 1.119	4.69 ± 1.401	5.64 ± 1.552	5.52 ± 1.030
	somatic symptom cluster	6.86 ± 1.612	7.25 ± 2.490	8.22 ± 1.606	7.43 ± 1.748
T3	psychological symptom cluster	7.85 ± 2.245	8.81 ± 3.146	9.31 ± 2.388	7.43 ± 1.964
	nutritional symptom cluster	6.01 ± 1.805	5.81 ± 2.136	6.44 ± 1.919	6.00 ± 1.483
	intestinal symptom cluster	5.72 ± 1.385	6.69 ± 2.798	6.83 ± 1.781	5.90 ± 1.261
	urinary system symptom cluster	3.00 ± 0.000	3.00 ± 0.000	4.78 ± 1.775	4.43 ± 1.207
	sexual life symptom cluster	5.67 ± 1.101	4.69 ± 1.401	5.61 ± 1.293	5.33 ± 1.017
	somatic symptom cluster	6.45 ± 1.664	7.00 ± 2.366	7.69 ± 1.411	6.90 ± 1.700
T4	psychological symptom cluster	6.94 ± 2.015	7.19 ± 3.868	7.19 ± 1.833	9.48 ± 2.600
	nutritional symptom cluster	4.71 ± 1.366	4.81 ± 2.228	5.06 ± 1.820	5.43 ± 1.777
	intestinal symptom cluster	5.35 ± 0.914	5.44 ± 1.504	5.33 ± 0.756	6.57 ± 2.271
	urinary system symptom cluster	3.00 ± 0.000	3.00 ± 0.000	3.50 ± 1.056	3.86 ± 1.878
	sexual life symptom cluster	5.45 ± 1.142	6.44 ± 1.365	5.28 ± 1.446	6.24 ± 0.995
	somatic symptom cluster	6.01 ± 1.482	6.56 ± 2.394	6.19 ± 1.064	7.90 ± 2.300

### 3.4 Analysis of influencing factors on symptom cluster trajectory categories in patients with concurrent chemoradiotherapy for cervical cancer

The univariate analysis of symptom cluster trajectory categories in patients with concurrent chemoradiotherapy for cervical cancer is as follows. There were statistically significant differences in trajectory categories among the four groups in terms of disease duration, surgical history, and presence of other chronic diseases (*P* < 0.05), as shown in **Table 4**.

**Table 4:** Univariate analysis of trajectory categories in patients with concurrent chemoradiotherapy for cervical cancer.

Project	Low symptom burden group (n=77)	Moderate symptom burden group (n=16)	High symptom burden group (n=36)	Moderate symptom burden- delayed symptom group (n=21)	Statistical value	*P*
Age					6.002^2)^	0.747
≤40	7(9.1)	1(6.3)	4(11.1)	3(14.3)		
41~50	7(9.1)	1(6.3)	5(13.9)	5(23.8)		
51~60	33(42.9)	9(56.3)	16(44.4)	6(28.6)		
>60	30(38.9)	5(31.3)	12(33.3)	7(33.3)		
Educational level					8.534^2)^	0.785
Primary school and below	51(66.2)	10(62.5)	22(61.1)	13(61.9)		
Junior high school	16(20.8)	5(31.3)	11(30.6)	5(23.8)		
High school or vocational school	7(9.1)	–	1(2.8)	2(9.5)		
College and above	3(3.9)	1(6.3)	2(5.6)	1(4.8)		
Occupation					20.340^2)^	0.200
Farmer	26(33.8)	5(31.3)	5(13.9)	5(23.8)		
Worker	1(1.3)	1(6.3)	4(11.1)	2(9.5)		
Enterprise employee	–	–	3(8.3)	1(4.8)		
Retired people	5(6.5)	–	3(8.3)	1(4.8)		
Freelance work	5(6.5)	1(6.3)	3(8.3)	2(9.5)		
Unemployed	40(51.9)	9(56.3)	18(50.0)	10(47.6)		
Number of children					3.421^2)^	0.752
0	3(3.9)	–	1(2.8)	2(9.5)		
1	22(28.6)	7(43.8)	18(50.0)	9(42.9)		
≥2	52(67.5)	9(56.3)	17(47.2)	10(47.6)		
Length pf marriage(year)					0.402^2)^	0.705
≤10	2(2.6)	–	2(5.6)	2(9.5)		
11-20	10(13.0)	1(6.3)	3(8.3)	2(9.5)		
>20	65(84.4)	15(93.8)	31(86.1)	17(81.0)		
Religion					0.746^2)^	0.917
No	72(93.5)	15(93.8)	34(94.4)	19(90.5)		
Yes	5(6.5)	1(6.3)	2(5.6)	2(9.5)		
Residence					10.311^2)^	0.101
Rural	16(20.8)	3(18.8)	3(8.3)	4(19.0)		
Villages and towns	10(13.0)	4(25.0)	10(27.8)	8(38.1)		
Urban	51(66.2)	9(56.3)	23(63.9)	9(42.9)		
Monthly household income(RMB)					4.312^2)^	0.319
<2000	15(19.5)	5(31.3)	7(19.4)	4(19.0)		
2000-5000	54(70.1)	10(62.5)	22(61.1)	16(76.2)		
>5000	8(10.4)	1(6.3)	7(19.4)	1(4.8)		
Medical expense payment method					9.851^2)^	0.052
Employee medical insurance	2(2.6)	–	2(5.6)	3(14.3)		
Residents’ medical insurance	75(97.4)	15(93.8)	34(94.4)	18(85.7)		
Commercial insurance	–	1(6.3)	–	–		
Tumor type					6.206^2)^	0.322
Squamous carcinoma	70(90.9)	13(81.3)	30(83.3)	17(81.0)		
Adenocarcinoma	3(3.9)	2(12.5)	5(13.9)	3(14.3)		
Other tumors	4(5.2)	1(6.3)	1(2.8)	1(4.8)		
Clinical stage					4.074^2)^	0.921
I	12(15.6)	3(18.8)	9(25.0)	4(19.0)		
II	27(35.0)	6(37.5)	9(25.0)	7(33.3)		
III	32(41.6)	6(37.5)	15(41.7)	10(47.6)		
IV	6(7.8)	1(6.3)	3(8.3)	–		
Courses of illness(month)					13.841^2)^	0.020
<3	13(16.9)	6(37.5)	1(2.8)	4(19.0)		
3~6	56(72.7)	9(56.3)	33(91.7)	13(62.9)		
>6	8(10.4)	1(6.3)	2(5.6)	4(19.0)		
History of surgery					12.171^1)^	0.006
No	39(50.6)	5(31.3)	27(75.0)	16(76.2)		
Yes	38(49.4)	11(68.8)	9(25.0)	5(23.8)		
Cobine with other diseases					11.699^1)^	0.008
No	40(51.9)	5(31.3)	26(72.2)	16(76.2)		
Yes	37(48.1)	11(68.8)	10(27.8)	5(23.8)		

1) χ^2^; 2) Fisher Exact Probability Method.

## 4 Discussion

### 4.1 Symptom clusters and their trajectory changes during concurrent chemoradiotherapy in cervical cancer patients

#### 4.1.1 Low symptom burden group

This study showed that 77 patients were in the low symptom burden group, accounting for the highest proportion (51.3%) of the total survey population, which is similar to the results of Lu Xiangyu (15) (52.91%). The characteristics of this group are that the psychological symptom burden level continued to decrease during the treatment period, the intestinal symptom group, nutritional symptom group, somatic symptom group, and sexual life symptom group gradually decreased after a slight increase at the end of 20 radiotherapy sessions, the urinary system symptom group had no symptoms throughout the course, and the overall symptom burden was at the lowest level compared with other categories three months after the end of radiotherapy. The patients in this category had a lighter symptom burden throughout the course, and the more severe emotional symptoms in the early stage could be relieved by themselves as the treatment progressed. The reason for this is that in the early stage of treatment, patients may be in a high degree of tension due to not having received comprehensive treatment, unfamiliarity with the environment, worries about physical damage, and concerns about high treatment costs. Studies have shown that radiotherapy itself and its complications can trigger anxiety in patients and exacerbate their psychological stress (16); mild to moderate self-perceived burden is common among cervical cancer patients receiving radiotherapy and is negatively correlated with quality of life (17, 18). Medical staff can intervene in the patient’s psychological state in the early stage before treatment to help the patient become familiar with the treatment environment and treatment process as soon as possible; inform the patient in advance of the possible side effects of treatment to reduce panic. During treatment, pay close attention to the patient’s emotional state and provide diversified psychological support: adopt cognitive behavioral therapy to improve negative cognition (19); relieve anxiety through mindfulness stress reduction training (20); combine traditional Chinese medicine emotional care and other characteristic techniques for comprehensive psychological adjustment (21); strengthen symptom management during treatment and reduce the psychological impact of side effects. For the low symptom burden group, the minimum intensity of intervention measures should be adopted, mainly focusing on observation and monitoring of symptoms. During follow-up visits, routine follow-up and appropriate health education are recommended.

#### 4.1.2 Moderate symptom burden group

The symptom burden group consisted of 16 patients, accounting for about 10.7% of the total population. The symptom burden of the psychological symptom group in this category showed a downward trajectory, but the decline was slightly smaller than that of the low symptom burden group; the nutritional symptom group and the sexual life symptom group followed similar trajectories to the low symptom burden group but had higher burden levels; the somatic symptom group had a low burden in the early stage, but showed signs of rebound three months after the end of radiotherapy; the urinary system symptom group had no symptoms. It is worth noting that the intestinal symptom group in this category was at a high level during radiotherapy. Gastrointestinal reactions caused by radiotherapy are relatively common in clinical practice. This is because the rectum is close to the uterus in the female anatomy and has a low tolerance to radiation. The specific manifestations are abdominal pain, diarrhea, constipation, hematochezia and other symptoms (22). Han Yufei’s study (23) showed that the incidence of radiation proctitis in cervical cancer radiotherapy patients was about 56.25%, and the probability of radiation proctitis increased with the patient’s age, the higher the tumor FIGO stage, the concurrent chemotherapy during radiotherapy, and the addition of lymph node dose. In addition, chemotherapy drugs (such as cisplatin) can inhibit the function of digestive glands and damage the digestive tract mucosa, further aggravating gastrointestinal symptoms such as nausea, vomiting, and loss of appetite. The research results of Lin Xuying (24) show that effective prevention and treatment measures can reduce the incidence of radiation enteritis to 2.31%. This suggests that medical staff should pay special attention to elderly and late-stage concurrent chemoradiotherapy patients, customize individualized vaginal radiation plans according to the patient’s tolerance, strengthen disease assessment and symptom observation, and use abdominal moxibustion, intestinal probiotics and other methods as adjuvant treatment. The moderate symptom burden group, while maintaining a state of low symptom burden during the later stages, should undergo symptom monitoring and routine follow-up; furthermore, appropriate self-management education can be provided to patients and their families to facilitate the early recognition of signs of relapse.

#### 4.1.3 High symptom burden group

The group of patients with a high symptom burden comprised 36 individuals, accounting for 24% of the total cohort. Their symptom burden peaked after 20 radiotherapy sessions and remained at a consistently high level throughout the entire treatment course; however, following the completion of treatment, their condition gradually improved after a period of rest. During treatment, patients in this category experienced a significant burden across various symptom clusters—specifically psychological, gastrointestinal, nutritional, and sexual health—which may potentially compromise their tolerance of and adherence to the prescribed therapy. Furthermore, it is noteworthy that these patients presented with relatively severe urinary tract symptoms. During concurrent chemoradiotherapy, patients typically experience a generalized decline in immune function; coupled with the susceptibility of the bladder to radiation-induced injury and the additional irritative effects of chemotherapy agents, the risk of urinary tract infections is substantially elevated. A study involving 204 patients undergoing radiotherapy for cervical cancer (25) reported an overall incidence of radiation cystitis of 32.4% following treatment, with mild-to-moderate forms of the condition predominating (31.4%). Given that radiation cystitis is often difficult to reverse once established, preventive measures are of paramount importance (26). Healthcare professionals should instruct patients to maintain a high fluid intake during treatment and to closely monitor the color and characteristics of their urine. Concurrently, clinicians should integrate clinical markers—such as leukocyte counts—into a comprehensive assessment to guide decision-making, administering anti-inflammatory medications as necessary to alleviate urethral pain. Moreover, nursing staff should prioritize their attention on this specific patient subgroup, ensuring early clinical identification and close observation. They should conduct periodic assessments of the patients’ physiological and psychological status and implement targeted management strategies, including stress-reduction techniques, dietary modifications, prophylactic pharmacotherapy, and education regarding sexual health preservation. During the post-treatment follow-up phase, the intervals between appointments should be shortened to closely monitor the patients’ recovery progress. When deemed necessary, patients should be referred to specialized services to facilitate a collaborative, multidisciplinary approach to intervention.

#### 4.1.4 Moderate symptom burden - delayed symptom group

This study showed that 21 patients (14% of all patients) had a moderate symptom burden-delayed symptom group. Their symptom burden was generally between that of the low and high symptom burden groups. However, three months after radiotherapy, their symptom burdens in the intestinal, somatic, psychological, and sexual symptom groups significantly increased. The symptom burdens in the nutritional and urinary tract symptom groups were also significantly higher than other categories, demonstrating significant population heterogeneity.

The manifestations of psychological symptom clusters may have a variety of complex causes: some patients adapt well to radiotherapy and are more sensitive to chemotherapy. The symptoms such as hair loss and vomiting caused by monthly chemotherapy significantly affect their emotional state; the side effects such as fatigue, loss of appetite and painful urination after radiotherapy persist; the sexual life between couples is affected by radiotherapy and has not recovered for a long time; it may also be related to the failure of the re-examination indicators to meet psychological expectations. It is recommended that nursing staff improve the continuity of care after treatment and conduct regular follow-ups to pay attention to the psychological changes of patients; the understanding and emotional support of the partner also play an irreplaceable role in the patient’s recovery process (27), and joint psychological counseling for couples can be considered in clinical work.

The burden of sexual symptoms in this group of patients peaks at T4. Studies have shown that cervical cancer patients commonly experience sexual dysfunction and low sexual satisfaction, leading to a decline in the quality of their sex life. These problems may be related to combined treatment (28). Radiotherapy can cause premature ovarian failure, which in turn leads to estrogen deficiency. The resulting changes in vaginal tissue can easily cause long-term sequelae, including vaginal dryness, fragility, and dyspareunia (29). Studies have shown that after pelvic radiotherapy, the incidence of vaginal stenosis in cervical cancer patients is about 79% (30). Medical staff should pay attention to the lag in the recovery of sexual function in cervical cancer patients: after radiotherapy and chemotherapy, patients often avoid sexual activity for fear of affecting recovery, and male partners may also reduce the frequency of sexual activity for fear of harming their partners or even fearing that they may also contract cancer; in addition, pain and bleeding caused by vaginal changes after radiotherapy further affect the recovery of patients’ sexual function. Studies have shown that the average time for cervical cancer patients to resume sexual activity is (6.6 ± 5.2) months (31). Therefore, trained specialist nurses are needed in clinical practice to provide professional guidance on sexual rehabilitation to help patients and their partners improve their understanding of the disease. Scholars both domestically and internationally recommend the use of vaginal dilators to effectively prevent and improve vaginal stenosis, typically starting about four weeks after the end of radiotherapy (32, 33). Simultaneous psychotherapy and adjunctive therapy are beneficial for promoting vaginal recovery and improving the quality of sexual life.

Studies have shown that the burden of somatic symptoms (including poor sleep, numbness in the hands and feet, pain, and fatigue) in this category of patients increases significantly 3 months after the end of treatment. Previous studies have shown (5) that 50% to 65% of cervical cancer chemotherapy patients still experience fatigue symptoms after treatment. Cancer-related fatigue is a persistent, painful, and subjective feeling of fatigue that is inconsistent with recent activity levels. Its occurrence may be related to the cancer itself or the treatment measures (27). Persistent fatigue can seriously affect the quality of life of cervical cancer patients after treatment and is not conducive to patient recovery (34). Clinical evidence shows that regular low- to moderate-intensity exercise during radiotherapy and chemotherapy is beneficial to the relief of fatigue (35). Medical staff should pay attention to patients’ related complaints, develop gradual activity plans for patients who can tolerate exercise, provide scientific exercise guidance, and assess reasonable exercise intensity.

The increased symptom burden at T4 in the symptom burden-delayed symptom group may be due to a variety of reasons: delayed response or cumulative effect of treatment-related toxicity; delayed symptom recognition caused by post-treatment recovery behavior; and exacerbation of real symptoms caused by poor disease control or disease progression. It should be noted that this study failed to collect specific information on treatment response, disease progression, and residual lesions at the T4 time point. This missing information may affect the interpretation of symptom trajectory. In addition to curing clinical symptoms, holistic care is the ultimate goal for cancer patients (36). Clinicians should pay close attention to these patients and be alert to the delayed symptom peak; it is recommended to increase the frequency of symptom screening and provide long-term support; conduct comprehensive health education and improve patients’ symptom self-management ability; risk classification can be carried out according to the specific situation of patients, and dynamic assessment can be performed through standardized scales during follow-up, and multidisciplinary cooperation can be used to promote patient recovery.

### 4.2 Factors influencing trajectory category in cervical cancer patients undergoing concurrent chemoradiotherapy

Univariate analysis showed that the course of the disease, whether surgery was performed, and whether other chronic diseases were present were influencing factors on the development trajectory of symptom clusters in different categories of cervical cancer patients (*P* < 0.05), which is similar to previous studies (37). It should be noted that this study only used univariate analysis to compare the differences in factors between different trajectory groups, without adjusting for potential confounding factors such as disease duration, surgical history, and chronic comorbidities through multivariate regression. The main reason for this is that some trajectory groups had small sample sizes and imbalanced class distributions; forcing multivariate analysis could lead to unstable model estimations, overly wide confidence intervals, or even complete dissociation or convergence failure. Therefore, the current results should be considered exploratory findings, and future studies should employ multivariate analysis with larger samples to control for confounding bias.

#### 4.2.1 Course of illness

The results of this study show that patients with a disease duration of >6 months are more likely to be classified as high symptom burden group. As the disease duration increases, cancer may spread to other organs and metastasize to distant sites. Long-term tumor consumption leads to decreased physical function and reduced tolerance to treatment (38). In addition, patients with a longer disease duration often undergo more treatments. However, the sequelae and delayed damage caused by multiple treatments can develop into chronic symptoms that are difficult to cure, leading to an increased symptom burden. Finally, the fear of disease recurrence and the economic pressure caused by treatment costs can lead to an increase in psychological problems such as anxiety and depression in patients (39). This suggests that clinicians should pay special attention to patients with a longer disease duration and provide emotional support while conducting timely symptom assessment and management.

#### 4.2.2 History of surgery

This study found that patients undergoing surgery were more likely to be classified as belonging to the high symptom burden group. Postoperative anatomical changes in cervical cancer can cause functional impairments, such as lower extremity lymphedema. The incidence of postoperative lower extremity lymphedema is as high as 69%, and it is currently incurable, severely impacting the quality of life of postoperative patients (40, 41). Postoperative sequelae can also overlap with treatment-induced symptoms, affecting the efficacy of subsequent treatments and increasing the physical burden on patients. In addition, the high symptom burden group still includes a high proportion of non-surgical patients, possibly due to confounding factors such as baseline disease symptoms or treatment indications. Therefore, healthcare professionals should closely monitor the symptoms and signs of postoperative patients and, when necessary, promote postoperative recovery through early intervention.

#### 4.2.3 Combine with other chronic diseases

This study shows that patients with other chronic diseases are more likely to belong to the high symptom burden group. These patients are generally in poorer physical condition and have lower treatment tolerance than the general population, and may experience overlapping symptoms leading to more complex symptoms and greater difficulty in symptom management. Interactions between anti-tumor drugs and medications for chronic diseases also increase the risk of adverse reactions. Healthcare professionals should pay close attention to the daily care and medication use of these patients, and strengthen symptom monitoring and prevention during treatment.

## 5 Conclusion

The study found that during concurrent chemoradiotherapy in cervical cancer patients, four different potential categories of symptom cluster trajectories could be identified: low symptom burden group (51.33%), moderate symptom burden group (10.67%), high symptom burden group (24%), and moderate symptom burden-delayed symptom group (14%). Significant differences existed among the different categories of patients in terms of disease duration, surgical history, and presence of other chronic diseases. These findings suggest that cervical cancer patients exhibit four symptom burden trajectory categories during concurrent chemoradiotherapy, and these are significantly associated with disease duration, surgical history, and presence of chronic diseases. Identifying different trajectory categories helps healthcare professionals develop more individualized symptom management strategies based on the dynamic characteristics of patients’ clinical symptoms.

Furthermore, this study has certain limitations. As a single-center survey, the sample size is small, and the study subjects are limited to a single tertiary-level hospital in Anhui Province, limiting the generalizability of the results. Future studies should expand the sample size and conduct multi-center studies, including patients from more regions and hospitals of different levels to further validate the findings. Secondly, the selected survey points in this study did not include baseline data, and the study primarily tracked different radiotherapy points. Future studies should compare symptoms with pre-treatment symptoms and include more key chemotherapy time points to more completely and comprehensively record changes in patients’ symptoms. Finally, the results are mainly based on patients’ self-reported subjective feelings, and the collected data may not be entirely objective and accurate. Future analyses can incorporate specific clinical indicators for further analysis.

## Data Availability

The raw data supporting the conclusions of this article will be made available by the authors, without undue reservation.
